# Defining characteristics and conservation of poorly annotated genes in *Caenorhabditis elegans* using WormCat 2.0

**DOI:** 10.1093/genetics/iyac085

**Published:** 2022-05-19

**Authors:** Daniel P Higgins, Caroline M Weisman, Dominique S Lui, Frank A D’Agostino, Amy K Walker

**Affiliations:** Program in Molecular Medicine, UMASS Chan Medical School, Worcester, MA 01605, USA; Lewis-Sigler Institute for Quantitative Genomics, Princeton University, Princeton, NJ 08540, USA; Program in Molecular Medicine, UMASS Chan Medical School, Worcester, MA 01605, USA; Department of Applied Mathematics, Harvard University, Cambridge, MA 02138, USA; Program in Molecular Medicine, UMASS Chan Medical School, Worcester, MA 01605, USA

**Keywords:** gene enrichment, function of unknown genes, *Caenorhabditis elegans*

## Abstract

Omics tools provide broad datasets for biological discovery. However, the computational tools for identifying important genes or pathways in RNA-seq, proteomics, or GWAS (Genome-Wide Association Study) data depend on Gene Ontogeny annotations and are biased toward well-described pathways. This limits their utility as poorly annotated genes, which could have novel functions, are often passed over. Recently, we developed an annotation and category enrichment tool for *Caenorhabditis elegans* genomic data, WormCat, which provides an intuitive visualization output. Unlike Gene Ontogeny-based enrichment tools, which exclude genes with no annotation information, WormCat 2.0 retains these genes as a special UNASSIGNED category. Here, we show that the UNASSIGNED gene category enrichment exhibits tissue-specific expression patterns and can include genes with biological functions identified in published datasets. Poorly annotated genes are often considered to be potentially species-specific and thus, of reduced interest to the biomedical community. Instead, we find that around 3% of the UNASSIGNED genes have human orthologs, including some linked to human diseases. These human orthologs themselves have little annotation information. A recently developed method that incorporates lineage relationships (abSENSE) indicates that the failure of BLAST to detect homology explains the apparent lineage specificity for many UNASSIGNED genes. This suggests that a larger subset could be related to human genes. WormCat provides an annotation strategy that allows the association of UNASSIGNED genes with specific phenotypes and known pathways. Building these associations in *C. elegans*, with its robust genetic tools, provides a path to further functional study and insight into these understudied genes.

## Introduction

Unbiased assays such as genetic screens, transcriptomics, and proteomics are powerful tools for identifying genes that are critical players in developmental processes, regulated in response to stress, or altered in disease processes. Recent technological improvements have vastly improved data quality and decreased the cost of transcriptomic analysis. Deep sequencing of mRNAs (RNA-seq) has, therefore, become a widely used assay to compare gene expression across cell types in differing physiological conditions or mutant backgrounds (Li *et al.* 2014). Proteomics approaches are also increasingly common and sophisticated and allow quantitative analysis of peptides present in specific subcellular compartments or carrying post-translational modifications (Fonslow *et al.* 2014). The genetic tools available in *Caenorhabditiselegans* complement these assays, as the results of -omics experiments can readily be subjected to functional validation and follow-up analyses. However, genome-wide -omics studies have some limitations as a discovery tool; unlike classical genetic approaches that allow the study of genes before the functions are known, -omics experiments depend on pathway analysis before the selection of genes for analysis. This directs focus toward well-studied genes and pathways. Indeed, multiple papers have noted that there is a selection toward analysis and publication on already well-studied genes (Hoffmann and Valencia 2003; Pandey *et al.* 2014; Haynes *et al.* 2018) and surprisingly, one study noted this effect increased over time (Haynes *et al.* 2018).

While -omics technologies can generate large amounts of high-quality data, several challenges complicate data interpretation, gene categorization, and data visualization. The GO (Gene Ontogeny) platform can be used to classify genes from -omics analysis according to biological or molecular function and cellular location. These analyses return categories that are statistically enriched in the input relative to the entire genome (Eden *et al.* 2009; Angeles-Albores, Robinson, *et al.* 2018; Mi *et al.* 2019). Other gene annotation databases, such as KEGG (Kyoto Encyclopedia of Genes and Genomes) provide pathway or enzymatic functional data (Thomas 2016). These resources contain valuable data; however, the highest-quality information is found for the most highly studied genes (Wood *et al.* 2019). Thus, they are of maximal utility in pathways that are already well-studied. Furthermore, uneven distribution of annotation terms introduces bias into statistical tests used for enrichment (Haynes *et al.* 2018). In addition, genes with unclear functions may be excluded from enrichment analysis (Ding *et al.* 2018). The genome-wide analysis also generates large and complex datasets, making intuitive data visualization an additional challenge. While bar charts that graph *P*-values are useful for visualizing single datasets, other styles such as bubble charts may be more useful for comparing data across multiple conditions.

*Caenorhabditis* *elegans* is amenable to genome-wide assays and multiple tools exist for pathway analysis of the results, most depending on GO annotation (Eden *et al.* 2009; Angeles-Albores, Lee, *et al.* 2018; Mi *et al.* 2019). To provide an easily visualized alternative to GO-based searches, we developed WormCat, a web-based program based on a near-complete annotation of the *C. elegans* genome (https://www.wormcat.com) (Holdorf *et al.* 2020). WormCat provides annotation for each input gene, determines category enrichment within the gene set, and provides scaled bubble charts for visualization. In the original version of WormCat, we included a category for poorly annotated genes (UNKNOWN) to avoid biasing the annotation lists toward well-studied genes. Using the whole-genome annotation list, we found that WormCat identified biologically significant categories from *C. elegans* exposed to RNAi or pharmacological treatments as well as from tissue-specific RNA-seq studies (Holdorf *et al.* 2020). In addition, we included annotation lists specific for the commonly used RNAi libraries and found these could reveal enriched pathways in data from an RNAi screen. WormCat has been rapidly adopted by the *C. elegans* community (Albarqi and Ryder 2021; Lee *et al.* 2021; Naim *et al.* 2021; Tecle *et al.* 2021; Zhang *et al.* 2021) and adapted into a metabolism-focused tool (Walker *et al.* 2021) as well as an integrated gene expression analysis program (Cheng *et al.* 2021).

The WormCat whole-genome annotation list contains 31,390 genes divided into nested categories (Supplementary Table 1), with 35 Category 1 (Cat1) groups. These Cat1 groups can be further divided into 242 Cat2 groups and then 361 Cat3 designations (see Supplementary Table 2 for category definitions). Each gene received a single Cat1, Cat2, and Cat3 classification. These were assigned hierarchically, based first on strict physiological functions (Holdorf *et al.* 2020). For example, NEURONAL FUNCTION (Cat1) contains genes that are shown to function only in neurons and does not contain genes that function in neurons as well as other cell types (Holdorf *et al.* 2020). Such pleiotropic genes, along with genes with defined biochemical functions, were placed in molecular-based categories. Next genes that lacked a clear molecular function but had specific and well-defined locations were placed in subcellular localization-based categories (Holdorf *et al.* 2020). Thus, the manually curated WormCat annotations are designed to provide distinct information from GO, utilizing the rich phenotypic data available in WormBase to provide high confidence gene assignments rather than potential functions. WormCat also classifies genes with little functional information as a separate category [UNKNOWN (WormCat 1.0) or UNASSIGNED (WormCat 2.0)]. Here, we focus on developing the UNASSIGNED category in WormCat to define characteristics of these genes and stimulate future functional studies. We find that representation of UNASSIGNED genes differs across tissues in published RNA-seq datasets. In addition, this category is poorly represented in multiple whole-animal proteomics datasets. By identifying enrichment and expression characteristics of genes with poorly defined functions, WormCat 2.0 will stimulate the inclusion of previously under-analyzed genes in functional studies. As 3% of poorly annotated genes have human orthologs, modeling analysis of unassigned genes in *C. elegans* provides a roadmap that can be used to extend the functional analysis of these genes. In addition, our analysis provides impetus to revisit poorly annotated gene across model organisms.

## Methods

### Annotations

WormBase version WS270 was utilized for WormBase descriptions (https://wormbase.org). We used ParaSite Biomart to obtain GO terms and predicted human orthologs for *C. elegans* genes (https://parasite.wormbase.org/biomart/martview). NCBI Conserved Domain Database (https://www.ncbi.nlm.nih.gov/Structure/cdd/wrpsb.cgi) and TOPCONS (https://topcons.cbr.su.se) were used for membrane-spanning domain predictions.

### abSENSE

abSENSE was downloaded from the publicly available github, https://github.com/caraweisman/abSENSE, and run locally. Run parameters were default. The input bitscore file was derived from BLASTP. The input evolutionary distance file was derived from 10 eukaryotic BUSCO genes determined to be single copy in all analysis species, which were then aligned using MUSCLE (default parameters), and used as input to the PROTDIST program (default settings) from the PHYLIP package.

### Scripts

WormCat 2.0 is built on the foundations of the original WormCat Web python codebase. A key new feature of Wormcat 2.0 is “Batch Processing,” which allows the execution of multiple datasets using a single submission. To support batch processing, we leveraged several open-source python packages and tools (Pandas, Celery, and Redis). The Pandas package handles data and file processing while Celery is used to queue and distribute the batch tasks, and Redis is the in-memory data structure store used as a message broker for Celery. All 3 of these technologies use the open-source BSD license.

## Results

### Evaluation of UNKNOWN/UNASSIGNED genes in WormCat 2.0

**Fig. 1.**
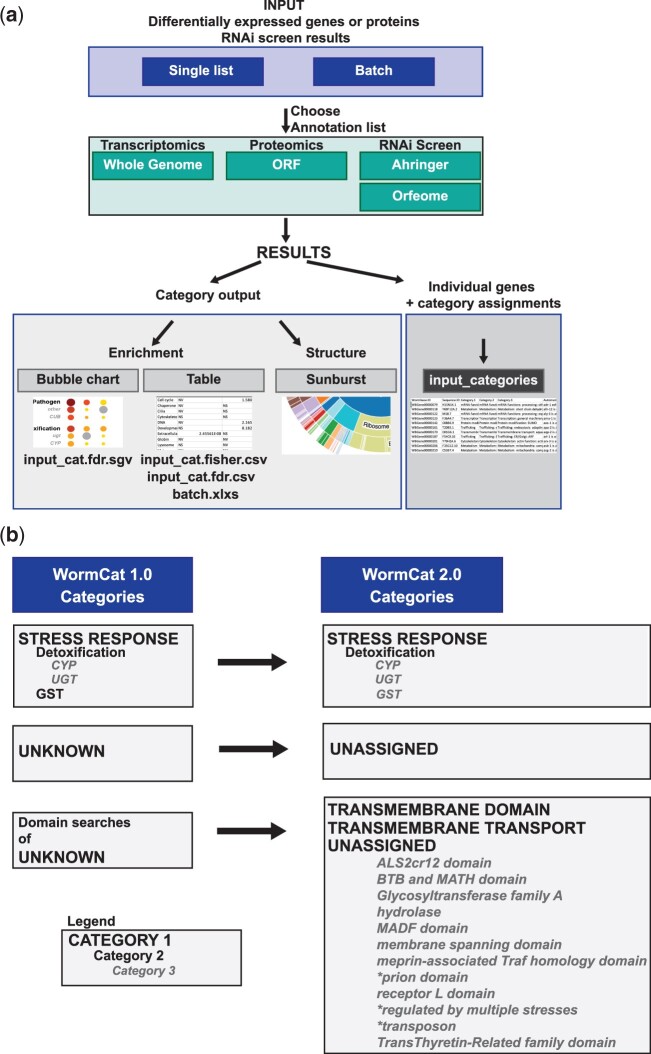
WormCat 2.0 supports category enrichment of multiple -omics datasets and allows identification of less well-characterized genes. a) Schematic diagram of WormCat 2.0 workflow. b). Changes in the annotation list structure from WormCat 1.0 to WormCat 2.0. See Supplementary Table 1 for annotation of each *C. elegans* gene, Supplementary Table 2 for annotation definitions, and Supplementary Table 3 for the list restricted to protein-coding genes. TM, transmembrane; CYP, cytochrome p450; CUB, complement C1r/C1s, Uegf, Bmp1; ugt, uridine diphosphate glucuronosyl transferase; GST, Glutathione-S-transferase.

WormCat is a program for pathway analysis of *C. elegans* RNA-seq, ChIP-seq, or genetic screen data that utilizes a near-complete annotation list of *C. elegans* genes (Holdorf *et al.* 2020). Input genes (regulated gene sets, RGS) entered as a single list or in a batch file are mapped to the specific annotation lists. Fisher’s exact test is used to determine the statistical significance of enrichment by building a contingency table that compares the number of genes in each category in the RGS to the entire annotation list along with a false discovery rate correction (see Fig. 1a). Because RNA-seq or ChIP-seq data contain information on noncoding RNA expression, the whole-genome annotation includes lincRNAs, miRNAs, snoRNAs, tRNAs, and pseudogenes. To enable category enrichment analysis of RNAi library screens, WormCat includes RNAi library-specific annotation lists to correct for the subgenomic size of the RNAi libraries (Holdorf *et al.* 2020). In addition, WormCat 2.0 provides an appropriate background ORF-only (open read frame) option for proteomics datasets (Fig. 1a). Category enrichment results from WormCat are provided as a scaled bubble chart (.sgv), a sunburst diagram, and .csv files. A gene-by-gene annotation of the input get is also provided. To facilitate the comparison of multiple datasets, we have added a batch processor also which produces a combined output file.

In WormCat 2.0, we used computational tools to rigorously examine protein domain-based annotations and continued to refine the annotation list to harmonize classification strategies. For example, in the “STRESS RESPONSE” category, we noted that Glutathione-S-Transferases (GSTs) were placed at a Cat2 level. In contrast, other enzymes involved in response to xenobiotics were in STRESS RESPONSE: Detoxification. We therefore reassigned the GSTs (Fig. 1b, Supplementary Tables 1 and 2). In the initial version of WormCat, we placed genes that did not meet annotation criteria for other categories in the “UNKNOWN” category so that statistical calculations included all genes, regardless of functional annotation. This category is now labeled “UNASSIGNED” to better reflect the common characteristic of this gene group (Supplementary Tables 1 and 2). Within Cat2 or Cat3, less well-characterized genes were also changed from “Cat2: other” to “Cat2: unassigned.” In the initial WormCat annotation list, 8,160 genes were classified as UNKNOWN, representing 26% of the *C. elegans* genome. Many of these genes had WormBase annotations with cellular locations or protein domains of unclear function (Harris *et al.* 2019). In our initial WormCat annotation list, we noted prion domains or induction of expression in response to multiple stresses at the Cat3 level. To expand these annotations, we used the KEGG database (Kanehisa and Goto 2000), NCBI Conserved Domain Database (Lu *et al.* 2020), TOPCONs membrane domain (Bernsel *et al.* 2009), and UNIPROT cellular localization predictions (Bateman *et al.* 2021) to examine each gene in the UNASSIGNED category (Supplementary Table 4). As a result, 81 genes were reassigned into Cat1 groupings such as “METABOLISM,” “STRESS RESPONSE,” or “mRNA FUNCTIONS” based on close inspection of homology-based UNIPROT annotation, or subcellular localization.

WormCat 1.0 placed membrane-spanning proteins with well-described functions into biological or molecular-based categories. Genes encoding transmembrane (TM) regions identified by the NCBI conserved domain database were placed in the “TRANSMEMBRANE DOMAIN” group (Holdorf *et al.* 2020). However, this excluded many genes with WormBase descriptions including the phrases “localizes to plasma membrane” or “ER protein” (Harris *et al.* 2019). To more rigorously identify TM proteins, we ran all the genes in the “UNASSIGNED” and “TRANSMEMBRANE DOMAIN” categories in the TOPCONS suite, which includes 6 algorithms for TM domain or signal sequence identification (Bernsel *et al.* 2009). We used the following criteria to assign categories (Fig. 1b, Supplementary Table 4). First, 41 genes with TM domains in all TOPCONs algorithms and domains common to transporters were reassigned to “TRANSMEMBRANE TRANSPORT.” Next, 1,466 genes showing predicted TM regions with all 6 programs in the suite, but lacking transporter-associated domains were placed in “TRANSMEMBRANE DOMAIN” (Fig. 1b, Supplementary Table 4). Next, those with TM regions predicted in 1 or 2 of the TOPCONS programs were placed in the UNASSIGNED: Unassigned: membrane-spanning domain. Finally, proteins with a predicted signal sequence but no TM domain were assigned to extracellular material: secreted protein. Analysis of the TRANSMEMBRANE DOMAIN proteins with TOPCON showed that 32 did not have 6/6 predictions from TOPCONS and were therefore moved to UNASSIGNED: Unassigned: membrane-spanning domain to denote lower confidence scores (Fig. 1b; Supplementary Table 4).

Some domain names, such as “BTB-POZ” or “Receptor L domain” identified in WormBase descriptions or the Conserved Domain Database, do not have precise functional characterizations, and others such as “hydrolase” may reflect a general enzymatic function, but the appropriate category is unclear. Therefore, we added 9 new Cat3 level annotations for multiple common but functionally unclear domains in the UNASSIGNED category. Based on these reannotations, 2,251 genes in UNASSIGNED were assigned to other categories or given additional category information at the Cat3 level. These annotation reassignments both improve the accuracy of WormCat and increase the curated information that can be applied to the UNASSIGNED genes to stimulate study and future improvements in functional information.

### Levels of lineage specificity in the UNASSIGNED gene category

**Fig. 2.**
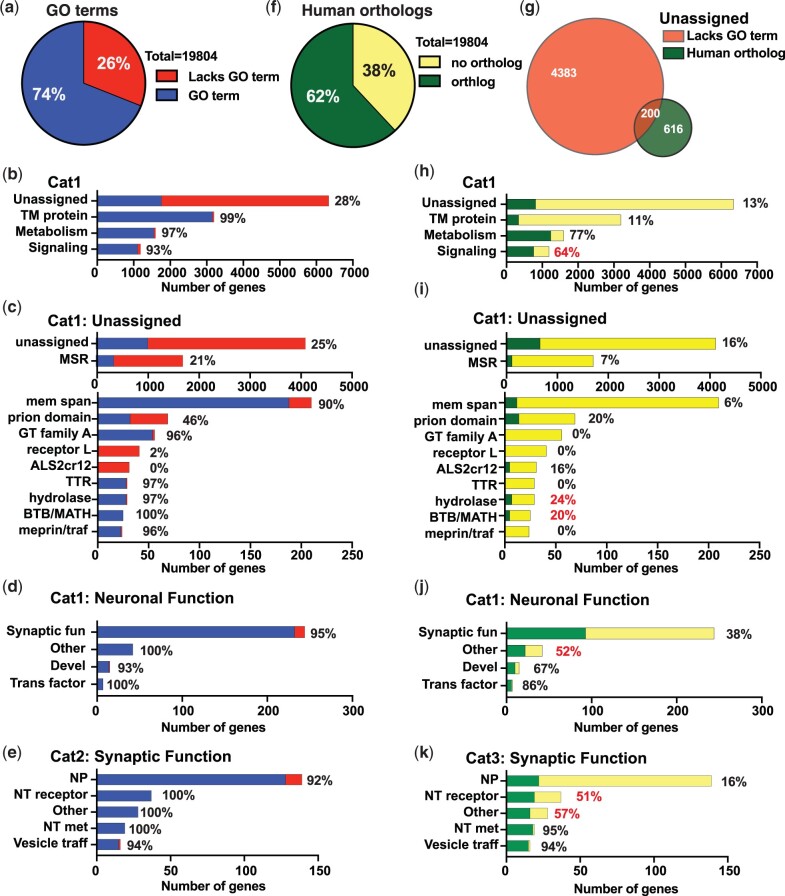
Unassigned genes in *C. elegans* include a subset with human orthologs. a) Pie chart of *C. elegans* protein-coding genes that are assigned GO terms in the ParaSite Biomart. b) Breakdown of WormCat Cat1 level categories with numbers of genes annotated by GO. Cat2 GO breakdown of Unassigned (c) and Neuronal function (d) with Cat3 level categories of Synaptic Function. Additional categories are in Supplementary Fig. 2, a–d. f) Pie chart of *C. elegans* protein-coding genes designated as having human orthologs in the ParaSite Biomart. g) Venn diagram showing the overlap between Unassigned genes lacking GO terms and those with human orthologs. h) Breakdown of WormCat Cat1 level categories with numbers of genes with human orthologs. Cat2 human ortholog breakdown of Unassigned (i) and Neuronal function (j) with Cat3 level categories of Synaptic Function (k) (Supplementary Fig. 2, e–h). Red percentages denote categories with substantially more human orthologs in a genome-wide BLASTP comparison of each *C. elegans* gene with the human genome (see Supplementary Table 4). TM, transmembrane; msr, multiple stress-regulated; mem span, membrane-spanning; GT family A, glucosyltransferase family A; TTR, TransThyretin-Related family domain; BTB/MATH, BR-C, ttk, and bab/meprin and TRAF homology; Synaptic fun, Synaptic function; Devel, Development; Trans, Transcription; NP, Neuropeptide, NT receptor, Neurotransmitter receptor; NT met, Neurotransmitter metabolism; Vesicle traff, Vesicle traffic.

One goal of providing curated information on UNASSIGNED category genes is to allow WormCat users to identify those with potential biological functions based on expression in RNA-seq, proteomic, or RNAi screen datasets. Like studies in mammalian systems (Hoffmann and Valencia 2003; Dolgin 2017; Haynes *et al.* 2018), we find that genes within well-studied areas such as NEURONAL FUNCTION and STRESS RESPONSE share high numbers of references and a rich subset of GO annotations (Supplementary Fig. 1, a and b). This is independent of the average number of genes in each reference or GO category (Supplementary Fig. 1, c and d). However, UNASSIGNED genes are poorly represented in the literature or by GO annotation. We next analyzed how many UNASSIGNED genes are annotated in GO, have human orthologs, or appear in lineage-specific gene families. Many unannotated genes are excluded from commonly used web-based programs, such GOrilla (Eden *et al.* 2009) for searching GO databases (Ding *et al.* 2018; Holdorf *et al.* 2020). In order to determine if the WormCat UNASSIGNED category overlaps with unannotated *C. elegans* genes in GO, we searched GO terms for each protein-coding WormBase ID in WormCat using the Parasite Biomart (Howe *et al.* 2017). We compared protein-coding genes in WormCat between genes outside the UNASSIGNED category (assigned) to those within it and found that while 26% assigned genes lacked associated GO terms, 72% of genes in the UNASSIGNED category were not annotated in GO. This makes the UNASSIGNED category the largest category of *C. elegans* genes that are not represented in GO (Fig. 2, a–e, Supplementary Table 4). Other WormCat categories with significant numbers of non-GO annotated genes include STRESS RESPONSE (11%), PROTEOLYSIS PROTEOSOME (20%), and NUCLEIC ACID (19%) (Supplementary Fig. 2a, Supplementary Table 4). Within the UNASSIGNED category, we annotated genes that were regulated in response to multiple stresses (MSR), contained a predicted TM domain, or domains of unclear function (Holdorf *et al.* 2020, see also Fig. 1b). While some of these categories are described in GO (membrane-spanning, TTR, hydrolase, and BTB/MATH), our annotation strategy separates higher or lower confidence TM proteins and allows identification of the MSR genes along with additional shared domain proteins (Fig. 2c, Supplementary Table 4).

Genes described as having human orthologs may be more extensively studied and better annotated (Wood *et al.* 2019). In order to determine if WormCat categories of genes lacking GO annotations were also predicted to lack human orthologs, we used the Parasite Biomart to obtain human ortholog predictions for each WormCat protein-coding gene. This website identified human orthologs for 62% of the *C. elegans* genes (Fig. 2, f and h–k, Supplementary Table 4) and is referenced to the current WormBase release in comparison to Ortholist (Kim *et al.* 2018). We noted that WormCat categories that were poorly annotated by GO also had low percentages of human orthologs (Fig. 2, b, c, h, and i, Supplementary Fig. 2, e–h, Supplementary Table 4). However, human orthologs were present in these categories, for example, the UNASSIGNED category contains 816 genes with human orthologs, 200 of which also lack a GO term (Fig. 2g). The human genome also contains many genes with poorly described functions, some of which may be linked to diseases through GWAS or disease-associated mutations (Haynes *et al.* 2018). In order to determine if any humans orthologs of the *C. elegans* UNASSIGNED genes were disease linked, we searched eDGAR, a database of human gene–disease associations (Babbi *et al.* 2017). eDGAR identified potential disease associations for 87 of these genes (Supplementary Table 4). For example, *tag-232* is closely related to human PACS1 (BLAST P *e*-value = 5.05E-67), which is associated with a form of a mental retardation Autosomal Recessive 43 (Supplementary Table 4). A small number of papers based on overexpression studies suggest a role in trafficking (Köttgen *et al.* 2005) and form the basis for UNIPROT annotation, however, rigorous functional studies have not been done (Riemann *et al*. 2017). Overall, this indicates that genes within the UNASSIGNED category have been understudied even though some have predicted orthology to human genes and could have disease relevance.

Other categories such as TM protein and STRESS RESPONSE were relatively well-annotated by GO but had fewer predicted human orthologs in the Parasite database (Fig. 2, b and h; Supplementary Fig. 2, a–d and e–g; Supplementary Table 4). As an alternative method of determining potential orthology, we performed BLASTP on each gene in WormCat. We found that around 2,800 genes that lacked human orthology annotation by Parasite Biomart had statistically significant *e*-values and had bit scores above 40 (Supplementary Table 4), suggesting that some of these genes could have human orthologs. However, the genes that were absent from the Biomart orthology database were largely from expanded gene families and were only 7% of the UNASSIGNED category (Fig. 2, i–k, Supplementary Table 4).

Many core-biological function genes are well conserved across phyla (Chervitz *et al.* 1998) and are extensively characterized. Sequence conservation between proteins is often determined by pairwise alignment determined by BLAST (Altschul *et al.* 1990) or with algorithms such as HMMR (www.hmmer.org). Genes that lack detectable homology by these methods are referred to as “lineage-specific,” often with the implication that they have specialized function (Cai *et al.* 2006). However, some of these genes may have structural conservation despite lacking sequence homology detectable by BLAST (Pearson 2013). Other genes may indeed encode proteins with lineage-specific functions or belong to classes of genes undergoing rapid evolution (Cai *et al.* 2006). For example, pathogen response genes may evolve rapidly to balance selection pressure (Sironi *et al.* 2015). In order to compare the number of lineage-specific genes in the UNASSIGNED category with other categories, we determined the number of species-specific and genus-specific genes (Zhou *et al.* 2015) defined by the absence of detected homologs in more distant species in each category (Supplementary Fig. 3, a–h, Supplementary Table 4). We found that many categories had percentages of nonlineage-specific genes that were close to numbers of human orthologs (Fig. 2, h–k, Supplementary Fig. 3, a–h, Supplementary Table 4), as might be expected. About half of the UNASSIGNED genes were found by Zhou *et al.* to be lineage-specific, with similar proportions in subcategories of completely undescribed genes or within the MSR subcategory (Supplementary Fig. 3c). Domain-defined subcategories had fewer lineage-specific genes. Lineage-specific genes were largely contained within the UNASSIGNED genes lacking human orthologs, defined by the Parasite Biomart (Supplementary Fig. 3d). However, our BLASTP results suggest some of these genes (bit score >40 and an *e*-value less than 0.01) contain sequence similarity to human genes (Supplementary Fig. 3j, Supplementary Table 4).

**Fig. 3.**
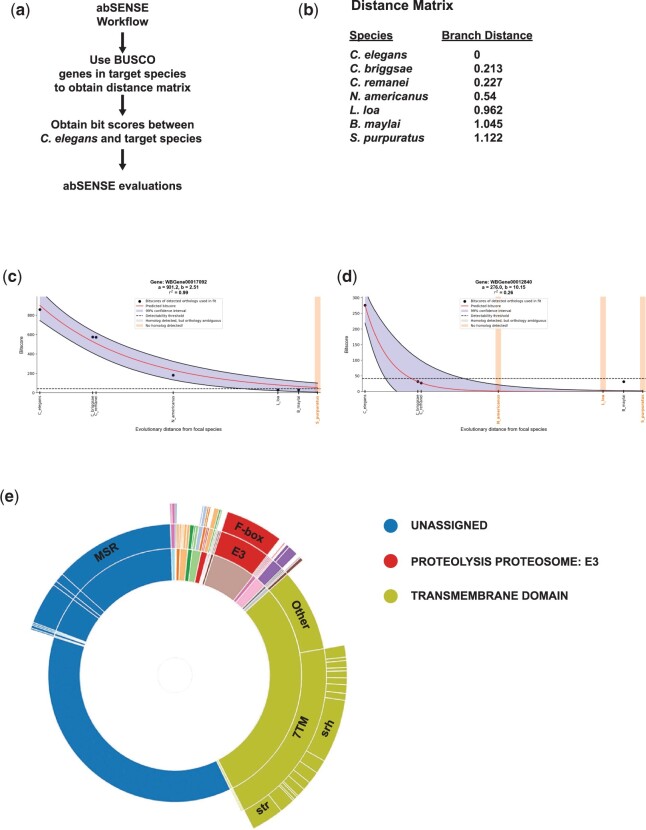
abSENSE analysis find many lineage-specificity of UNASSIGNED could be overestimated. a) Schematic showing abSENSE workflow. b) Distance matrix of species used in analysis. Example Venn diagrams (c, d). e) WormCat sunburst showing categories of *C. elegans* genes unlikely to have homologs in *S. purpuratus.*

It is also possible that proteins have evolutionarily conserved functions, but the amino acid similarity is not apparent by BLAST or other algorithms (Pearson 2013). A recent study has developed a phylogenetic method (abSENSE) to assess whether a gene could be lineage-specific for this reason. This method uses distance matrixes to predict the likelihood that orthologs in outgroups could be undetectable by BLAST (Weisman *et al.* 2020). Using yeast and *Drosophila*, this study found that many genes considered to be lineage-specific arise from a failure to detect homology using present methods and also identified gene homologies that require an explanation beyond homology detection failure. This latter class was highlighted as particularly interesting candidates for functional studies. We adapted this method to estimate if any of the genes in the UNASSIGNED category could have undetected orthologs outside *Caenorhabditis* compared to the lineage-specific genes defined by Zhou *et al.* abSENSE makes predictions for whether a gene may have a homolog in a target species based on the evolutionary distances between that target species and the focal species (Weisman *et al.* 2020). We chose to include *C. elegans* and target species comprising 2 sister species, *Caenorhabditisremenai*, and *Caenorhabditisbriggsae*, along with Clade III nematodes, *Necator americanus*, *Loa loa*, and *Brugia maylai*, and so calculated evolutionary distances between these species (Fig. 3, a and b). We also included a more distantly related invertebrate, the sea urchin *Strongylocentrotus purpuratus*. Most UNASSIGNED genes had orthologs in some of these target species, suggesting that they are not specific to *Caenorhabditis* (example in Fig. 3c, Supplementary Table 4). Other genes were specific to *Caenorhabditis*; among these, we identified cases in which any potential homologs outside of the genus are likely to be undetectable (Fig. 3d). For these genes, homologs may indeed be present in outgroup species: in this scenario, they appear lineage-specific merely because these homologs have diverged too far to be detected by standard homology search. Finally, we also identified cases in which potential homologs outside the genus, specifically in *S. purpuratus*, would likely have been detected if present, implying that they may be truly specific to the *Caenorhabditis* genus. We used WormCat to classify these potentially novel genes and found that the majority were in the UNASSIGNED, TRANSMEMBRANE: 7TM and PROTEOLYSIS: E3:Fbox family (Fig. 3e, Supplementary Table 4), which are examples of rapidly expanding gene families in *C. elegans* (Robertson and Thomas 2006).

### UNASSIGNED: regulated by multiple stress genes show *pmk-1* and *atf-7*-dependence

**Fig. 4.**
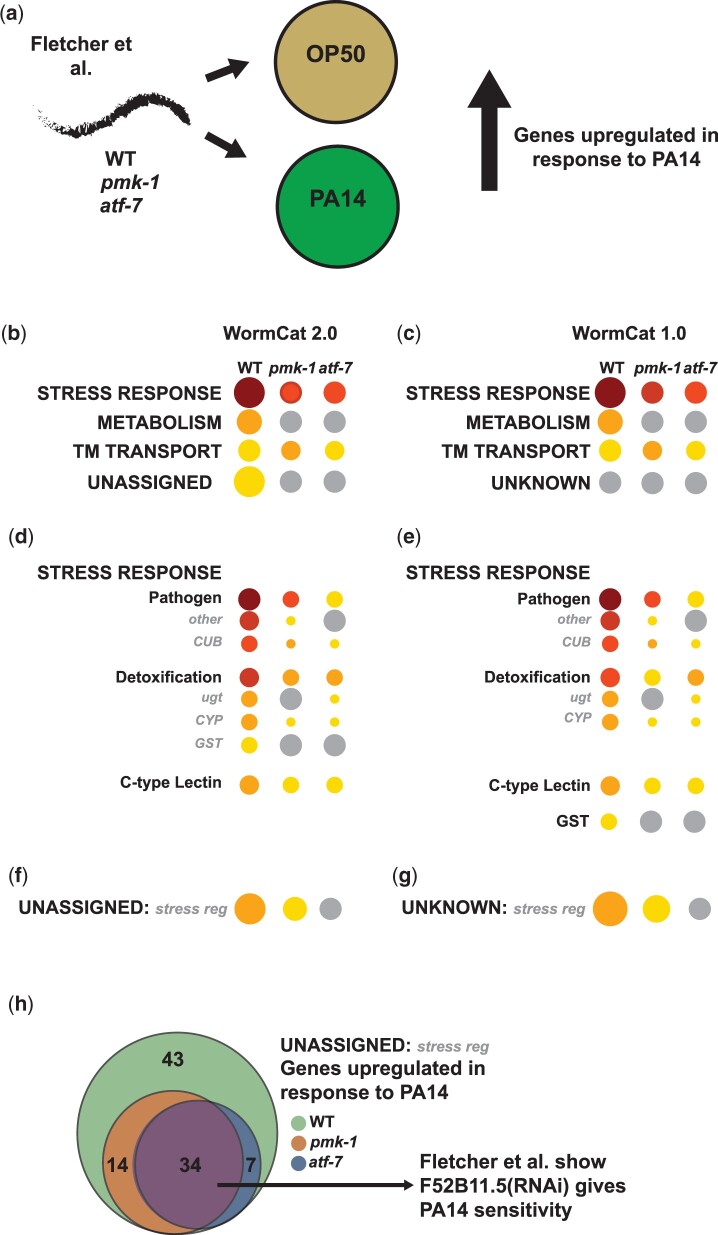
UNASSIGNED: regulated by multiple stress genes are regulated by a *pmk-1/atf-7* immunity circuit in response to *P. aeruginosa* (PA14). a) Schematic diagram showing experimental workflow from Fletcher *et al.* investigating gene regulation in response to PA14. Cat1 category enrichment in WormCat 2.0 (b) compared with WormCat 1.0 (c). Cat2 level enrichment of Stress Response and Unassigned/Unknown in WormCat 2.0 (d, f) and WormCat 1.0 (e, g). h) Venn Diagram showing dependence on *pmk-1* and *atf-7* of UNASSIGNED genes upregulated by PA14. See also Supplementary Table 5. TM, transmembrane; CYP, cytochrome p450; CUB, complement C1r/C1s, Uegf, Bmp1; ugt, uridine diphosphate glucuronosyl transferase; GST, Glutathione-S-transferase.

Annotation changes in WormCat 2.0 altered multiple categories, including the STRESS RESPONSE, TRANSMEMBRANE PROTEIN, TRANSMEMBRANE TRANSPORT, and UNASSIGNED categories. In order to test how WormCat2.0 performs, we determined gene enrichment on published RNA-seq data that examined which *Pseudomonas aeruginosa* (PA14) upregulated genes were also dependent on the MAP Kinase *pmk-1*/MPK13 and the bZIP transcription factor *atf-7* (Fletcher *et al.* 2019; Fig. 4a). Both WormCat 2.0 and 1.0 show highly significant enrichment in STRESS RESPONSE: Pathogen and Detoxification (Fig. 4, b and c; Supplementary Table 5), consistent with the authors’ GO enrichment findings as well as previous results (Troemel *et al.* 2006; Ding *et al.* 2018; Fletcher *et al.* 2019). The STRESS RESPONSE: Detoxification category has a slightly higher enrichment in WormCat 2.0 since GSTs are included at the Cat3 level (Fig. 4, d and e). Reannotation of the UNASSIGNED genes also added genes to the TM TRANSPORT category. However, we do not find significant changes in enrichment in TM TRANSPORT in this gene set from WormCat 1.0 to 2.0 (Fig. 4, a and b; Supplementary Table 5). The “UNASSIGNED: regulated by multiple stresses” category is enriched in *C. elegans* exposed to PA14 in this study and a previous version using WormCat (Ding *et al.* 2018; Fletcher *et al.* 2019; Fig. 4, b and c; Supplementary Table 5). The UNASSIGNED: regulated by multiple stresses genes (MSR genes) lack characteristics that allow assignment into physiological or molecular categories but had changes in expression due to at least 2 common stress agents such as paraquat, methylmercury, or tunicamycin (see Holdorf *et al.* 2020, for a complete list).

Interestingly, many of the MSR genes upregulated in response to PA14 are also dependent on *atf-7* and the *pmk-1* pathway (Fig. 4, f–h; Supplementary Table 5). Fletcher *et al.* (2019) tested 45 PA14 upregulated/*atf-7*-dependent genes for PA14 sensitivity and found 14 survived less well on pathogenic bacteria (Esp phenotype). Many of these genes with the Esp phenotype contained domains known to be important for pathogen responses. However, the authors found an Esp phenotype for one ATF-7-target with no known domains (Fletcher *et al.* 2019). This gene, F52B11.5, is a WormCat MSR gene and is also regulated by methylmercuric chloride, Cry5B, and *hif-1/HIF1*. Thus, genes within the MSR gene category appear to be regulated similarly to genes in well-described pathogen responses, including genes with critical biological responses. By defining a category for enrichment, WormCat provides a framework for future studies of genes that would ordinarily be overlooked for further analysis because less is known about their function.

### Tissue-specific expression of UNASSIGNED genes

**Fig. 5.**
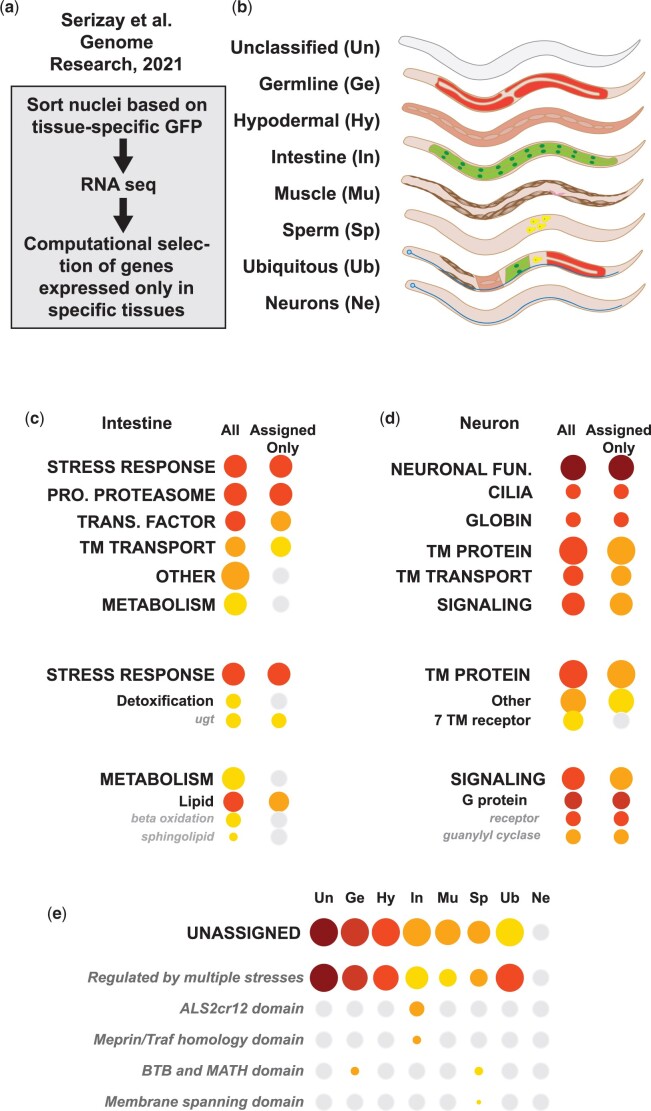
Tissue-specific RNA-seq shows enrichment of UNASSIGNED genes in datasets specific to multiple cell types, but not neurons. a) Schematic diagram showing workflow from (Serizay *et al*. 2020)*.* Comparison of category enrichment with inclusion (All) and exclusion (Assigned only) of UNASSIGNED genes in Intestine-only (b, c) and Neuron-only (b, d) genes. e) Cat3 breakdown of UNASSIGNED genes in Unclassified (Un), Germline (Ge), Hypoderm (Hy), Intestine (In), Muscle (Mu) Sperm (Sp), and Neuron-only genes as in (b). See also Supplementary Tables 4 and 6. Pro, Proteolysis; Trans., Transcription; TM, transmembrane; ugt, uridine diphosphate glucuronosyl transferase.

Identification of category enrichment commonly depends on Fisher's exact test, a statistical metric that builds a contingency table to determine the likelihood that the number of items in the group in a test set is more enriched than the number of those items in the entire set. Thus, enrichment statistics can be affected by the number of items in the entire set. We sought to explicitly test the hypothesis that excluding genes based on annotation status altered the statistical metrics using a tissue-specific RNA-seq dataset published by the Ahringer lab (Serizay *et al*. 2020). We determined category enrichment RNA-seq data from 2 tissues, Intestine-only and Neurons-only, and compared 2 versions of the WormCat annotation list, All (including UNASSIGNED genes) and Assigned only. While the top categories of STRESS RESPONSE, PROTEOLYSIS PROTEOSONE, TRANSCRIPTION FACTOR, and TRANSMEMBRANE TRANSPORT remained significant, METABOLISM failed significance at the FDR correction (Fig. 5b, Supplementary Fig. 4, a and b; Supplementary Table 6) although the *C. elegans* intestine has a clearly defined role in metabolism (McGhee 2007; Dimov and Maduro 2019). At the Category 2 level, removing the UNASSIGNED genes alters the enrichment of detoxification genes in the STRESS RESPONSE category for intestinally expressed genes; however, METABOLISM: Lipid remains statistically enriched (Fig. 5b, Supplementary Fig. 4, a and b; Supplementary Table 6).

On the other hand, UNASSIGNED genes are not enriched in Neuronal-only RNA-seq datasets, and the major enriched Cat1 groups all remain statistically significant (Fig. 5, b and c; Supplementary Fig. 4c; Supplementary Table 6). At the Cat2 level, removing the UNASSIGNED genes shifts the 7TM protein out of the significance range, but SIGNALING Cat2 groups remain significant. Taken together, this evaluation shows that excluding unannotated genes has different effects on pathway analysis of RNA-seq from distinct tissues. Thus, the UNASSIGNED gene category has an important role in stabilizing effects on annotation bias.

The observation that there were different numbers of UNASSIGNED genes in Intestine-only vs Neuronal-only tissues in the (Serizay *et al*. 2020) data prompted us to examine the enrichment of UNASSIGNED genes in other tissues in this dataset. Strikingly, Neuronal-only tissue is the only group that lacks enrichment of UNASSIGNED genes (Fig. 5d; Supplementary Table 6). This appears to be largely driven by the UNASSIGNED MSR genes. One explanation for the difference in tissue distribution of the UNASSIGNED genes could be that genes expressed in neurons are more likely to be conserved, well-studied, and better annotated. In order to address this question, we examined the numbers of GO annotated genes and predicted human orthologs in our NEURONAL FUNCTION category. These genes were defined as having curated functions in neurons that were not shared with other tissues, and NEURONAL FUNCTION is enriched in multiple independent tissue-specific datasets (Holdorf *et al.* 2020; Fig. 5d). WormCat NEURONAL FUNCTION genes were well-annotated by GO, and about half the genes had predicted human orthologs in the Parasite Biomart (Fig. 2, d and e; Supplementary Tables 2a and 6). However, nuclei isolated from neurons by (Serizay *et al*. 2020) show enrichment in categories with low numbers of human orthologs, such as TM transport and TM protein (Figs. 2i and 5d); therefore, there may be other explanations for the apparent tissue-specificity of the UNASSIGNED genes.

### UNASSIGNED genes are poorly enriched in proteomics samples

**Fig. 6.**
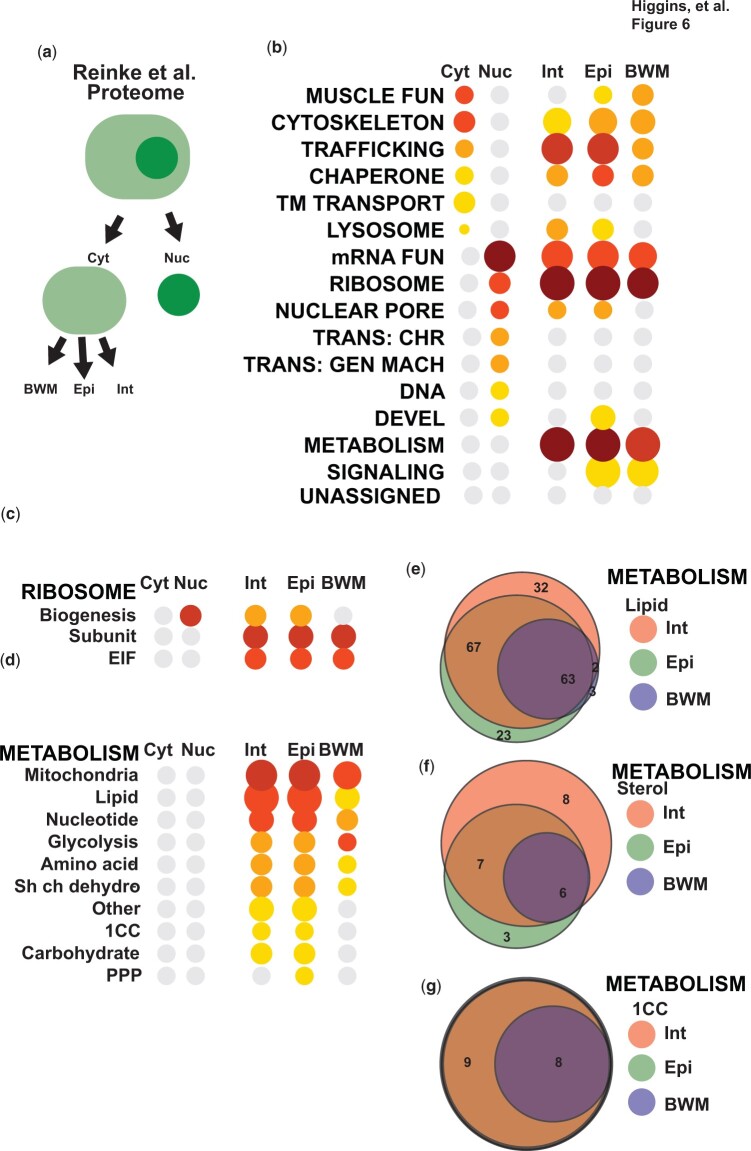
Tissue and compartment-specific proteomic data lack enrichment in UNASSIGNED genes. a) Workflow for Reinke *et al.* b) Cat1 level enrichment of proteins in cytoplasmic or nuclear extracts along with cytoplasmic extracts from Intestine (Int), Epidermis (Epi), or Body Wall Muscle (BWM). Breakdown of WormCat enrichment in Ribosome (c) or Metabolism (d). Venn diagrams showing the number of tissue-specific genes in METABOLISM (e), METABOLISM: Lipid (d), or METABOLISM: 1CC. See also Supplementary Fig. 5 and Supplementary Table 7. Cyt, Cytoplasmic; Nuc; Nuclear, Trans., Transcription; TM, transmembrane; EIF, elongation and initiation factor; 1CC, 1-carbon cycle; PPP, pentose phosphate pathway.

The annotation lists in WormCat were designed for whole-genome RNA-seq or ChIP-seq assays and so included both protein-coding and noncoding genes (Holdorf *et al.* 2020). To provide an appropriate annotation list for proteomics, we removed nontranscribed genes (ORF-only annotation list). This annotation list retains the category of NON-CODING RNA; however, the genes that remain function in processing noncoding RNAs (Supplementary Table 1). Next, we examined gene enrichment patterns in 2 published proteomics datasets (Narayan *et al.* 2016; Reinke *et al.* 2017). Reinke *et al.* (2017) performed proteomics on nuclear vs cytoplasmic fractions as well as obtaining nuclear and cytoplasmic proteomes from multiple tissues (see Fig. 6a). Enrichment of mRNA functions, NUCLEAR PORE, and the Transcription-associated categories in the nuclear fractions was expected; however, RIBOSOME was strongly enriched (Fig. 6b; Supplementary Table 7). As ribosomes are localized to the cytoplasm, we examined the gene enrichment patterns at the Cat2 level and found the major enrichment in the nuclear samples was in RIBOSOME: biogenesis, which is a nucleolar process (Fig. 6c; Supplementary Table 7). RIBOSOME: Subunit and RIBOSOME: EIF were strongly enriched in the cytoplasmic fractions of intestinal, epidermal, and body wall muscle cells. Comparison of category enrichment between cell types did not reveal large differences at the Cat1 level (Fig. 6b; Supplementary Table 7). Both the intestine and epidermis play important roles in *C. elegans* metabolism. Detailed examination of these proteomes also showed similar patterns (Fig. 6c; Supplementary Table 7). We next asked if the same proteins were driving enrichment in the METABOLISM subcategories or if there were tissue-specific differences. While many of the proteins in the category “METABOLISM: Lipid” were shared, some were tissue-specific (Fig. 6e; Supplementary Table 7). The Cat3 group with the most differences was METABOLISM: Lipid: sterol (Fig. 6f; Supplementary Table 7). The group includes many short-chain dehydrogenases that can contribute to hormone production (Mahanti *et al.* 2014) and thus have may tissue-specific functions. In contrast, the 1-Carbon cycle (1CC) genes detected in the proteomics were the same in the intestine and epidermis (Fig. 6g; Supplementary Table 7).

In contrast to multiple tissue-specific RNA-seq datasets (Fig. 5; see also Holdorf *et al.* 2020), the UNASSIGNED category is not enriched in any of the samples in the Reinke *et al.* data. In order to verify this in a different proteomics dataset, we used the ORF annotation list in WormCat to find enriched categories in proteomics comparing young adult and aging worms (Narayan *et al.* 2016). The authors also performed fractionations separating cytoplasmic, membrane-bound, and nuclear proteins (Supplementary Fig. 5c and Supplementary Table 8). Because of the lower number of detected membrane-bound and nuclear proteins, we limited our search for enriched categories to “all detected,” “changed in aging,” and the “cytoplasmic fraction.” WormCat finds strong enrichment of METABOLISM, mRNA FUNCTIONS, and RIBOSOME in the cytoplasmic category, whereas enrichment DNA functions and NUCLEAR PORE are lost in comparison to All Detected (Supplementary Fig. 5d and Supplementary Table 8). METABOLISM: Lipid and STRESS RESPONSE are enriched in proteins that change during aging (Supplementary Fig. 5d and Supplementary Table 8). Similar to the proteomics data from Reinke *et al.*, there is no enrichment for the UNASSIGNED category. As the authors of Reinke *et al.* point out, proteomics is less sensitive than RNA-seq, and several studies have pointed out a discordance between mRNA and protein expression (Harvald *et al.* 2017). Thus, proteins in the UNASSIGNED group could be lower than the threshold for proteomics detection.

## Discussion

### Strategies for study of poorly annotated genes

Fully sequenced genomes for the major model systems as well as humans have been available for 20 or more years (Gates *et al.* 2021). However, each of these genomes contains large numbers of genes that are poorly annotated. For example, the human genome contains around 20,000 protein-coding genes, and 3,000 have limited functional annotations (Dey *et al.* 2015). Even well-characterized eukaryotes with smaller genomes, such as *Saccharomyces cerevisiae* contain large numbers of genes with unknown functions (Wood *et al.* 2019). These genes may be understudied due to research bias if no obvious human ortholog or disease relevance is evident. The biological functions of these genes may be elusive because of functional redundancy or because they function in specific contexts that are difficult to replicate in experimental environments. One approach to providing functional information on these genes is to determine mRNA expression patterns and define coexpression groups (Pertea *et al.* 2018) to identify processes that might be shared among the coexpressed genes. Other studies have used protein:protein interactions defined by either yeast 2-hybrid (Y2H; Rolland *et al.* 2014) or mass spec (Huttlin *et al.* 2017) to link poorly annotated proteins to well-described processes. Indeed, Roland and Vidal found that the accumulation of Y2H data increased study of and publications on individual genes (Rolland *et al.* 2014). Thus, providing interaction or coexpression data on poorly annotated genes can spur studies that provide insight into biological function.

The *C. elegans* genome contains around 19,000 protein-coding genes (Schwarz 2005). Large-scale RNAi and mutant screens ascribe phenotypes to around one-third of the genome (Schwarz 2005), and other genes may be annotated based on homology to human genes (WormBase). However, 26% of the genomes lack GO annotation (Fig. 2a). Early estimates projected that 9% of the *C. elegans* genome is conserved across metazoa, yet the functions of these genes are unknown (Schwarz 2005). The exclusion of poorly annotated genes not only biases statistical metrics used in enrichment analysis (see Fig. 4, Supplementary Fig. 5, and Supplementary Table 5) but also discourages further study of these genes. By improving annotation of UNKNOWN/UNASSIGNED genes, the analysis provided by WormCat 2.0 allows these genes to appear in enrichment analysis. Using an RNA-seq dataset exploring the bacterial pathogen response (Fletcher *et al.* 2019), we found that more than half of the enriched UNASSIGNED: multiple stress-regulated genes were regulated by the same signaling and transcriptional network that controlled the canonical stress response (Fig. 3). Interestingly, the authors found that one of these genes affected survival upon PA14 exposure (Fletcher *et al.* 2019), demonstrating biologically relevant activities in this gene set.

The genes in the UNASSIGNED set have a higher proportion that lack GO terms, and many appear to lack human orthologs or are lineage-specific. Some of these genes may have homology that is missed due to structural similarity that is not reflected at the amino acid level or because homology is undetectable by BLAST (Pearson 2013). We used abSENSE, a recently developed tool that uses evolutionary distances to determine whether a gene could appear lineage-specific merely because of failure to detect homologs in outgroups. We found that homology detection failure could be sufficient to explain lack of orthologs in other *Caenorhabditis* species, the clade III nematodes *Brugia malayi* and *L.loa* and a non-nematode invertebrate, the echinoderm *S.purpuratus*. We found that most UNASSIGNED genes were found to have orthologous proteins in *S. purpuratus* and so were not lineage-specific. For others, however, homology detection failure appeared to be a plausible explanation for the apparent lineage-specificity. This result indicates that the number of lineage-specific genes may be overestimated (Weisman *et al.* 2020). UNASSIGNED genes that appear *C. elegans*-specific may not be selected for functional testing as they lack definable human disease or functional relevance. It is striking that the UNASSIGNED genes are underrepresented in neuronal cells in published tissue-specific RNA-seq data from (Serizay *et al*. 2020) (see Figure 5). This could be an artifact of our annotation strategy; however, it might also reflect tighter evolutionary constraints in these specialized cells.

### Strategies for category enrichment

The tractability and affordability of -omics technologies have allowed *C. elegans* researchers to compare whole-genome mRNA or protein distributions between mutant or RNAi backgrounds and a wide variety of environmental conditions. These studies then rely on category enrichment tools to identify genes for further analysis. We have extensively compared WormCat to GO-based enrichment tools (Holdorf *et al.* 2020) and found that WormCat identifies biologically relevant gene sets not revealed in GO. However, alternative category enrichment tools employ distinct strategies, and dataset analysis may benefit from cross-platform analysis. For example, EVITTA, a web-based tool for RNA-seq analysis, allows downloads from GEO and enrichment by KEGG, GO, or WormCat term along with several alternatives for visualization (Cheng *et al.* 2021). The Kenyon lab developed the Gene Modules tool, which integrates gene coexpression data (Cary *et al.* 2020). Functional characterization of poorly annotated genes is a complex problem that may require multiple strategies to reveal gene–phenotype connections, utilizing multiple platforms for gene pathway analysis. WormCat 2.0 provides a platform that allows characterization of understudied genes. Thus opening up a previously enigmatic group of *C.elegans* genes for further study.

## Data availability

###  

WormCat: The WormCat 2.0 code is available under MIT Open-Source License and can be downloaded from the GitHub repository https://github.com/dphiggs01/wormcat along with all annotation lists and version-control information. WormCat can also be installed R package using the devtools library for direct usage. Wormcat 2.0 may be accessed at www.wormcat.com.

Supplementary material is available in figshare: https://doi.org/10.25386/genetics.19755412abSENSE: The abSENSE code is available on Github: https://github.com/caraweisman/abSENSE. This code was published by Weisman *et al.* (2020).The data analyzed in this study has been published in the following papers: Fletcher *et al.* (2019), Serizay *et al.* (2020), Reinke *et al.* (2017), and Narayan *et al.* (2016).

## Supplementary Material

iyac085_Supplementary_Data
