# Supplementary material for: Defining characteristics and conservation of poorly annotated genes in Caenorhabditis elegans using WormCat 2.0
Source: Genetics. 2022 May 19;221(4):iyac085. doi: 10.1093/genetics/iyac085 (PMC9339291; doi:10.1093/genetics/iyac085)
Supplement: iyac085_Supplementary_Data [file iyac085_supplementary_data.docx]

**Supplemental Material Legends**

**Supplemental Figure 1: Neuronal function and stress response categories show rich GO annotation and citation numbers. A.** Average number of references listed in WormBase or GO terms provided by Parasite for genes in Neuronal Function, Stress response or Unassigned Categories. **B.** Descriptive statistics for (A) from Graphpad Prism. **C**. Number of genes per reference or GO term as in **A. D**. Descriptive statistics for (**C**) from Graphpad Prism.

**Supplemental Figure 2: Unassigned genes in *C. elegans* include a subset with human orthologs. A**. Breakdown of WormCat Cat1 level categories with numbers of genes annotated by GO. Cat2 GO breakdown of TM protein/Signaling (**B**) and Stress Response at the Cat2 (**C**) and Cat3 level (**D**). **E**. Breakdown of WormCat Cat1 level categories with numbers of genes designated as having human orthologs in the ParaSite Biomart. Cat2 human ortholog breakdown of TM protein/Signaling (**F**) and Stress Response at the Cat2 (**G**) and Cat3 level (**H**). Red percentages denote categories with substantially more human orthologs in a genome-wide BLASTP comparison of each *C. elegans* gene with the human genome (see **Supplemental Table 4**). Abbreviations: TM, transmembrane; Stress resp, Stress response; Prot prot, Proteolysis Proteasome; Trans factor, Transcription factor; EC, Extracellular; mRNA fun, mRNA function, Prot general, Proteolysis General; Neuro fun, Neuronal function; Protein mod, Protein modification; Devel, Development; Trans chr, Transcription Chromatin; Trans. GM, Transcription: General Machinery; Sig. GPCR, Signaling: G-protein coupled receptor; CUB, CUB, complement C1r/C1s; NLP, neuropeptide-like protein; CYP, cytochrome p450; ugt, uridine diphosphate glucuronosyl transferase; GST, Glutathione S-transferase.

**Supplemental Figure 3:** **Unassigned genes in *C. elegans* include predicted lineage-specific and non-lineage-specific genes. A**. Pie chart of *C. elegans* protein-coding genes predicted to be lineage-specific by Zhou et al (Zhou *et al.* 2015). **B**. Breakdown of WormCat Cat1 level categories with numbers of predicted lineage-specific genes. **C.** Cat2 GO breakdown of Unassigned genes**. D.** Venn Diagram illustrating the number of lineage-specific genes and those with no predicted human ortholog within the UNASSIGNED gene set. Predicted lineage-specific gene number Cat2 level categories in Neuronal Function (**E**) and Cat3 level categories in Synaptic Function (**F**). Breakdown of numbers of lineage-specific genes within the Cat2 level of TM protein/Signaling (**G**) and Stress Response at the Cat2 (**H**) and Cat3 level (**I**). (See **Supplemental Table 4**). Abbreviations: TM, transmembrane; msr, multiple stress-regulated; mem span, membrane-spanning; GT family A, glucosyltransferase family A; TTR, TransThyretin-Related family domain; BTB/MATH, BR-C, ttk, and bab/meprin and TRAF homology; Synaptic fun, Synaptic function; Devel, Development; Trans, Transcription; NP, Neuropeptide, NT receptor, Neurotransmitter receptor; NT met, Neurotransmitter metabolism; Vesicle traff, Vesicle traffic; Stress resp, Stress response; Prot prot, Proteolysis Proteasome; Trans factor, Transcription factor; EC, Extracellular; mRNA fun, mRNA function, Prot general, Proteolysis General; Neuro fun, Neuronal function; Protein mod, Protein modification; Devel, Development; Trans chr, Transcription Chromatin; Trans. GM, Transcription: General Machinery; Sig. GPCR, Signaling: G-protein coupled receptor; CUB, CUB, complement C1r/C1s; NLP, neuropeptide-like protein; CYP, cytochrome p450; ugt, uridine diphosphate glucuronosyl transferase; GST, Glutathione S-transferase.

**Supplemental Figure 4: Contingency tables comparing the significance of Cat1 level categories with and without UNASSIGNED genes.** Data tables showing numbers of genes and enrichment values when all genes (**A, C**) are used rather than functionally annotated genes only (Assigned only) (**B, D**) in the intestine (**A, B**) and Neuron (**C, D**). the p-value is Fisher's Exact Test; gray highlighting shows values that are below an FDR of 0.01. Abbreviations: TM, transmembrane; RGS, Regulated Gene Set.

**Supplemental Figure 5** **Tissue and compartment-specific proteomic data lack enrichment in UNASSIGNED genes. A.** Workflow for Reinke et al. **B**. Cat1 level enrichment of proteins in cytoplasmic or nuclear extracts along with nuclear extracts from Intestine (Int), Epidermis (Epi), or Body Wall Muscle (BWM). **C**. Workflow schematic for Narayan, et al. D. Cat1 level enrichment of proteins from all conditions, those that are changing in aging animals (Δ Aging) and from Cytoplasmic extracts. See also **Supplemental Table 7, Supplemental Table 8**. Abbreviations: TRANS., transcription; PROT., proteolysis; SILAC, Stable Isotope Labeling by/with Amino acids.

**Supplemental Table 1:** **WormCat annotations**. xlsx file containing annotation definitions.

**Supplemental Table 2: WormCat annotation definitions.** xlsx file containing annotation definitions. Tabs: 1. Cat1 definitions, 2. Cat2 definitions, 3. Cat3 definitions. Color key: light blue highlight, other to assigned; light yellow highlight, new category; border, substantial change of genes in a category.

**Supplemental Table 3:** **WormCat ORF annotations**. xlsx file containing annotation definitions for protein-coding genes.

**Supplemental Table 4:** **GO terms and human orthologs for WormCat genes**. xlsx file matching WormCat protein-coding genes to GO terms and human orthologs. Tabs: 1. GO_human_ortholog from, 2. human.bit_abSENSE, 3. Unassigned_GoRILLA, 4. GoRILLa_listed_gene.GO.

**Supplemental Table 5: WormCat output for Fletcher at al.**. xlsx file comparing WormCat 2.0 and WormCat 1.0 annotation lists. Blue shading show significance level. Tabs: 1. WC2.0_Cat1, 2. WC2.0_Cat2, 3. WC2.0_Cat3, 4. N2_up_WC2.0_cat, 5. up_pmk-1_WC2.0_cat6. up_atf-7_WC2.0_cat, 7. N2_down_WC2.0_cat, 8. down_pmk-1_WC2.0_cat, 9. down_atf-7_WC2.0_cat, 10. WC1.0_Cat1, 11. WC1.0_Cat2, 12. WC1.0_Cat3, 13. N2_up_WC1.0_cat, 14. up_atf-7_WC1.0_cat, 15. up_pmk-1_WC1.0_cat, 16. N2_down_WC1.0_cat, 17. down_pmk-1_WC1.0_cat, 18. down_atf-7_WC1.0_cat.

**Supplemental Table 6: WormCat output for Serizayat al.**. xlsx file comparing WormCat 2.0 annotation lists with and without UNASSIGNED genes. Blue shading shows the significance level. Tabs1. WC2.0_all_Cat1, 2. WC2.0_all_Cat2, 3. WC2.0_all_Cat3, 4. WC2.0_Assingned_Cat1, 5. WC2.0_Assingned_Cat2, 6. WC2.0_Assingned_Cat3, 7. Contingency_All, 8. Contingency_intestine, 9. Contingency_neurons.

**Supplemental Table 7: Protein category enrichment in Reinke et al**. xlsx file containing WormCat output for proteomics using the ORF only annotation list. Blue shading shows the significance level. Tabs: 1. Cat1, 2. Cat2, 3. Cat3, 4. Cytoplasm-specific genes,5. Nucleus-specific genes,6. Body wall muscle (BWM) cytoplasmic genes,7. Epidermis cytoplasmic genes,8. Intestine cytoplasmic genes,9. Body wall muscle (BWM) nuclear genes,10. Epidermis nuclear genes, 11. Intestine nuclear genes.

**Supplemental Table 8: Protein category enrichment in Narayan et al**. xlsx file containing WormCat output for proteomics using the ORF only annotation list. Blue shading shows the significance level. Tabs: 1. Cat1, 2. Cat2, 3. Cat3, 4. all_detected_peptides_cat, 5. aging.change_cat, 6. Cytoplasm_cat.
